# Leishmaniasis: a review

**DOI:** 10.12688/f1000research.11120.1

**Published:** 2017-05-26

**Authors:** Edoardo Torres-Guerrero, Marco Romano Quintanilla-Cedillo, Julieta Ruiz-Esmenjaud, Roberto Arenas

**Affiliations:** 1 Sección de Micología, Hospital “Manuel Gea González” Secretaría de Salud, Calz. de Tlalpan 4800, Ciudad de México 14080, Mexico; 2 Dermatólogo, Clínica Carranza, Chetumal, Quintana Roo, Mexico

**Keywords:** Leishmaniasis, Leishmania, cutaneous-chondral, chicleros ulcer

## Abstract

Leishmaniasis is caused by an intracellular parasite transmitted to humans by the bite of a sand fly. It is endemic in Asia, Africa, the Americas, and the Mediterranean region. Worldwide, 1.5 to 2 million new cases occur each year, 350 million are at risk of acquiring the disease, and leishmaniasis causes 70,000 deaths per year. Clinical features depend on the species of *Leishmania *involved and the immune response of the host. Manifestations range from the localized cutaneous to the visceral form with potentially fatal outcomes. Many drugs are used in its treatment, but the only effective treatment is achieved with current pentavalent antimonials.

## Introduction

Leishmaniasis is a tropical and subtropical disease caused by an intracellular parasite transmitted to humans by the bite of a sand fly, mainly *Phlebotomus* and *Lutzomyia* (Europe, Northern Africa, the Middle East, Asia, and part of South America); exceptionally, transmission has also been reported as a laboratory accident ^1^. According to the World Health Organization (WHO), leishmaniasis is one of the seven most important tropical diseases and it represents a serious world health problem that presents a broad spectrum of clinical manifestations with a potentially fatal outcome ^2, 3^. It is found in all continents except Oceania ^2, 4^ and is endemic in circumscribed geographic areas in Northeastern Africa, Southern Europe, the Middle East, Southeastern Mexico, and Central and South America.

The clinical features include a broad range of manifestations with different degrees of severity that depend on the species of *Leishmania* involved and the immune response of the host ^3^. In Mexico, the most characteristic form is the cutaneous-chondral form ^1, 4^, also called “chiclero’s ulcer”.

## Epidemiology

Leishmaniasis is a disease with a worldwide distribution; it is found in about 89 countries ^4, 5^. It is endemic in Asia, Africa, the Americas, and the Mediterranean region. In the American continent, it is mainly a jungle zoonosis (but it can be acquired in semi-desert or cold regions) transmitted by sand flies mainly of the genera *Phlebotomus* and *Lutzomyia*. It is found in many countries, from the southern United States to the northern provinces of Argentina (seroprevalence of 0.17% for cutaneous leishmaniasis [CL]) ^6^ with the exceptions of Chile, Uruguay, and El Salvador ^4, 7, 8^.

Between 12 and 15 million people in the world are infected, and 350 million are at risk of acquiring the disease. An estimated 1.5 to 2 million new cases occur each year, and it causes 70,000 deaths per year ^4, 9^.

In 2012, the WHO led an effort to report on the burden and distribution of leishmaniasis in 102 countries, areas, or territories worldwide—to identify cases of CL and visceral leishmaniasis (VL)—and the data until 2010 indicate that 90% of global cases of VL occurred in Bangladesh, Brazil, Ethiopia, India, South Sudan, and Sudan and that about 70% of CL cases occurred in Afghanistan, Algeria, Brazil, Colombia, Costa Rica, Ethiopia, Iran, Sudan, and the Syrian Arab Republic.

On the other hand, of 25 countries selected to study the burden of this disease, 13 have a high burden of VL (Bangladesh, China, Ethiopia, Georgia, India, Kenya, Nepal, Paraguay, Somalia, South Sudan, Spain, Sudan, and Uganda), 11 have a high burden of CL (Afghanistan, Algeria, Colombia, Iran, Morocco, Pakistan, Peru, Saudi Arabia, Syrian Arab Republic, Tunisia, and Turkey), and one (Brazil) has a high burden of both clinical forms ^10^. In Turkey, around 2,000 autochthonous cases of CL are reported each year.

Owing to the civil war in Syria, Turkey received around 3 million refugees, and they are located mainly at either camps or homes in the south or southeastern part of Turkey. An epidemiological study conducted in Turkey from 2009 to 2015 with 263 patients positive for leishmaniasis using real-time and semi-nested polymerase chain reactions (PCRs) revealed that 66.15% were Turkish, 33.46% were Syrian, and 0.38% were Afghani; the species detected were *Leishmania tropica* and *Leishmania infantum* (from Turkish and Syrian patients), demonstrating the effects of the war in that territory ^11^.

Khezzani and Bouchemal, in Algeria, reported 4,813 confirmed cases of CL in a period of 13 years: the disease affected all municipalities and all age groups ^12^. The most affected individuals were 10 to 19 years old (31.41%) and children less than 9 years old (25.70%). Most of the patients were males (65%).

In Iran, Holakouie-Naieni *et al*. identified the geographic and seasonal distribution of CL: there were 589,913 cutaneous cases, the annual incidence was approximately 30.9 per 100,000 and most cases occurred in the central regions of the country ^13^, and the highest prevalence rate (35.14%) of lesions occurred in autumn ^14^.

VL, also known as kala-azar, is a clinical form endemic in Bangladesh, India, and Nepal. More than 60% of all VL cases worldwide correspond to South Asia, with a predominance in rural areas. In this respect, in Nepal, approximately 5.7 million people are considered to be at risk and are confined mainly to the Terai region, which borders the VL-endemic districts of the Bihar state in India. Moreover, from 1980 to 2007, a total of 23,368 cases, including 311 deaths, were reported. According to the annual health report of the Department of Health Services in Nepal, the incidence of VL was 2.67 per 10,000 people at risk in 2006–2007 ^15^.

In Latin America, it is estimated that around 60,000 new cases (including all types) occur each year. The disease is typical of environments with an altitude of 0 to 1,500 m above sea level, temperatures higher than 20°C, and an annual rainfall of 1,500 to 3,000 mm. However, some cases known locally as “Uta” have been documented in the highlands of Peru, which are cold and humid places where the disease is transmitted by a small insect ^16, 17^.

More commonly, it affects men, and in Mexico the main risks are seen among farmers (gum tree harvesters, cocoa and banana farmers, among others), loggers, hunters, precious timber exploiters, military personnel, biologists, ornithologists, and those who practice ecological tourism; it affects the collectors of yerba mate (tea), rubber, banana, coffee, and coca in Peru and Brazil ^1, 17, 18^.

**Figure 1.**
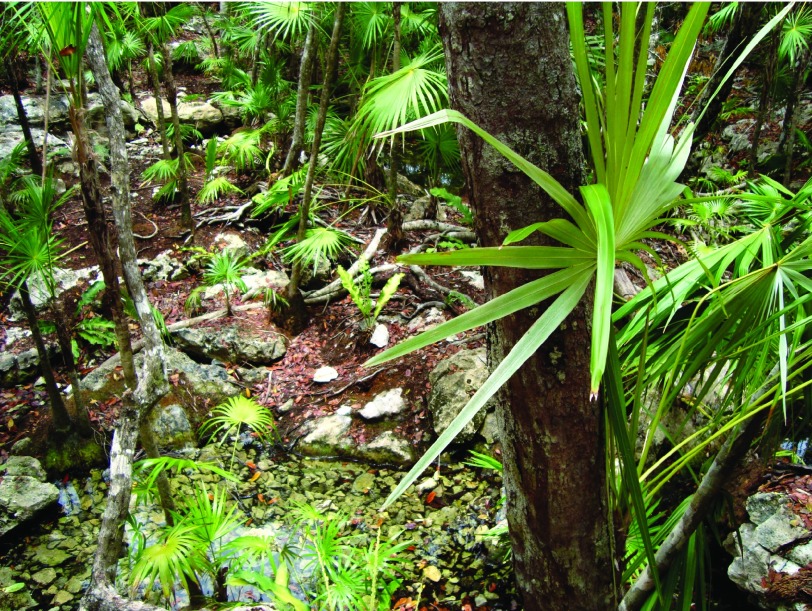
Rainforests where chewing gum workers and farmers extract sap from *Manilkara zapota* trees (chewing gum tree).

Women, the elderly, and children are also at risk of exposure in endemic zones. In Mexico, the disease has been known since the pre-Hispanic era ^18^, and all of the clinical forms of leishmaniasis have been reported ^19^. The most common presentations are the pure cutaneous and the cutaneous-chondral forms, which when affecting the ear cause the classic chiclero’s ulcer (gum tree harvester’s ulcer). The main endemic area in this country is found in the Neotropical zone from the southeast (southern Veracruz, Tabasco, Oaxaca, Chiapas, Campeche, Quintana Roo, and part of Yucatán) ^1, 2, 7, 18, 20^ ( Figure 1). In this country, an annual incidence of 5.08 cases per 100,000 population has been observed in the Yucatán Peninsula ^21, 22^. Serological studies carried out in Becanchén (southern state of Yucatán) have documented specific antibodies in 17% of the general population. Also, in Tabasco, an incidence and prevalence rate of 2.35 and 9.41 per 100,000 population, respectively, have been observed; therefore, it is considered a high-endemic zone ^18^. These rates are explained because farmers work only 10 km from the site in which the greatest quantity of rodents’ reservoirs and transmission vectors are found ^23^. Positive serology is observed in 20.42% of military personnel working in this zone ^24^. The two main vectors of this disease in the Yucatán Peninsula are *Lutzomyia olmeca* and *Lutzomyia cruciate* ^23, 25^. In Campeche and Yucatán (Mexico), *Leishmania mexicana* is the main causative agent; *Leishmania braziliensis* predominates in Belize and Guatemala, where cases of *L. mexicana* have also been reported.

In an epidemiological study in Matto Grosso (Brazil), Thies *et al*. showed that the frequency and diversity of sand flies were higher in rural areas with no deforestation (areas of permanent preservation [96.85%]) than in urban areas ^26^. *Lutzomyia dasypodogeton* was the most frequent species in areas of preservation, *Lutzomyia antunesi* was more frequent in neighborhoods with forest fragments and semi-urban areas, and *Lutzomyia aragaoi* was the most frequent species in completely urbanized neighborhoods; 87.92% of frequency and diversity of sand flies was observed in the rainy season versus 12.08% in the dry season.

In a descriptive study conducted by Vita *et al*., supported by data from the Brazilian Information System of Notifiable Diseases (SINAN) and the Brazilian Institute of Geography and Statistics (IBGE), 1,470 cases of CL were analyzed; 87.89% were of the cutaneous clinical form, and there were higher incidences in the white ethnic group (49.72%) and in the 20- to 39-year-old age group (32.44%) ^27^. These researchers concluded that there was a decrease in the number of cases based on a coefficient of detection from 1.44 per 100,000 inhabitants in 2004 to 0.20 per 100,000 in 2013.

In an epidemiological and historic study in Palestine, Al-Jawabreh *et al*. identified *Leishmania* species by using amplification products from a PCR-internal transcribed spacer 1 (PCR-ITS1) followed by restriction fragment length polymorphism (RFLP) analysis using Hae III and showed that the case numbers peaked in 1995, 2001, 2004, and 2012 ^28^. Statistically significant clusters of cases caused by *Leishmania major* were restricted to the Jericho District, and those caused by *L. tropica* were located in the districts of Jericho, Bethlehem, Nablus, and Tubas.

In an epidemiological study in Afghanistan, Fakhar *et al*. confirmed 3,861 cases of CL in a period of 1 year, and the predominant species of all molecularly identified samples using ITS1 PCR-RFLP analysis was *L. tropica* (98%) ^29^.

CL is found in Southern Europe, Asia (the Middle East, Afghanistan, and Pakistan), Africa, and Latin America. On the other hand, the cases of cutaneous disseminated leishmaniasis have been described in the northeast of Mexico and in states neighboring the southern United States and in the state of Texas ^7, 20^.

Muco-cutaneous leishmaniasis is found in Latin America and less frequently in Africa (Ethiopia and Sudan). VL, or kala-azar, is found in Northern and Eastern Africa and in Northern Asia (India, Pakistan, Bangladesh, Nepal, and China), in Southern Europe, and in Latin America ^8^. Bangladesh, Bhutan, India, and Nepal represented more than 73% of the global burden of VL in 2012, and the prevalence of confirmed post–kala-azar dermal leishmaniasis ranged between 4.4 and 4.8 per 10,000 population in Bangladesh and India ^30^.

Transmission of this disease is low in deforested areas, and the only isolated species in those habitats has been *Lutzomyia deleoni*, which is not an anthropophilic species ^25^. Nevertheless, since 1960, Forattini has emphasized the potential capability of vectors to adapt to suburban and urban environments ^7^. Important contributors for the spread of leishmaniasis include environmental factors such as alterations in temperature and water storage, irrigation patterns, deforestation, climate changes, immunosuppression by HIV, organ transplant or immunotherapy, development of drug resistance, increased traveling to endemic regions, dog importation, wars, natural disasters, and communities with poor socioeconomic status ^31^.

Despite its great epidemiological importance, leishmaniasis is considered by the WHO to be a disease that has been neglected by different public and private organizations financing health improvement and research ^1, 4^.

As part of the adaptation phenomenon of vectors to semi-urban and urban environments, domestic animals play a role as new opportunistic hosts ^4^. A hypothesis that would explain the new epidemiological dynamics is the progressive destruction of the vector’s natural habitat by humans, natural disasters, and global warming. The populations at risk are defined by patterns of human travel related to tourism and by populations displaced by wars, economic crises, and natural disasters ^4^. In this regard, the case of Afghanistan is particularly telling. In its capital, Kabul, there were practically no reported cases of leishmaniasis before 1990; however, by 2003, 25,000 cases were documented and treated. Newer data have indicated 67,500 new cases per year ^4, 8^.

## Etiopathogenesis

The putative vectors of the different species and subspecies of protozoa of the *Leishmania* genus are dipterans of the genus *Lutzomyia* in the New World and *Phlebotomus* in the old continent belonging to the subfamily Phlebotominae. The taxonomy of these vectors is as follows:

Kingdom     Animal

Phylum        Arthropoda

Subphylum   Euarthropoda

Superclass    Antenata

Class            Insecta/Hexapoda

Order           Diptera

Suborder      Nematocera (considered the most ancient members, dating to the Jurassic period)

Family          Psychodidae

Subfamily     Phlebotominae

Genus          Old World: *Phlebotomus/Sergentomyia*

                    New World: *Lutzomyia* (with six subgenera: *Lutzomyia*, *Dampomyia*, *Pintomyia*, *Nyssomyia*, *Psychodopygus*, and *Peruensis/Brumptomyia/Warileya/Psychodopygus*)

Species *Olmeca*

*Flaviscutellata*

*Trapidoi*

*Diabolica*

*Longipalpis*

The genus *Lutzomyia* includes 90% of the pathogenic species, most of which infect humans. *Lutzomyia* is present in the tropics of the New World. They display a dorsal hump and wings with a lanceolate oval shape. Only females nourish from blood, usually from mammals but sometimes also from inferior terrestrial vertebrates. Usually they nourish at night; during the day, they hide in dark, humid places. *Lutzomyia* absorb sugars that may have an important role in the development of *Leishmania* in the vector species ^7^.

Laison and Shaw proposed a classification of leishmaniasis on the basis of their pattern of development in the bowel of the sand fly, which not only assumes evolution of the parasites but also allows their classification into groups such as suprapillary, peripillary, and hypopillary. In the hypopillary group, infection is limited to the hindgut (that is, pylorus ileum and rectum). The reservoir’s hosts are limited to reptiles from the Old World: *Leishmania agamae* and *Leishmania ceramodactyli*. The peripillary group requires mandatory development of the parasite in the hindgut but migrates to the midgut and foregut. The reservoir hosts are reptiles in the Old World ( *Leishmania adleri* and *Leishmania tarentolae*) and mammals in the New World ( *L. braziliensis* complex). In the suprapillary group, development does not occur in the hindgut, and the parasites are restricted to the midgut and foregut. The host reservoirs are mammals in the Old as well as in the New World (species: *tropica*, *major*, *donovani*, *mexicana*, and *hertigi*). Therefore, those parasites are grouped into two subgenera: *Leishmania*, which includes parasites that develop in the midgut and foregut, and *Viannia*, which develops in the *Phlebotomus* foregut, midgut, and hindgut.

The subgenus *Viannia* (Laison and Shaw, 1987) includes the following: *L. braziliensis*, which is the most frequent causative agent of cutaneous and muco-cutaneous clinical forms; *Leishmania guyanensis*, whose geographical localization corresponds to the north of the Amazon River, the Guianas, Venezuela, and Peru and which is usually the cause of multiple cutaneous lesions; *Leishmania lainsoni*, which is limited almost exclusively to the Brazilian Amazon; *Leishmania shawi*, which is found in the Brazilian state of Pará; *Leishmania naiffi*, which is distributed in Pará and Amazonas (Brazil) and in the French Guiana and is responsible for the cutaneous form of the disease; *Leishmania peruviana*, which is found mostly in the Peruvian Andes and causes the CL form known locally as “Uta”; *Leishmania panamensis*, which is the responsible causative agent of the disease in Panama, Costa Rica, Colombia, Ecuador, and Honduras; and *Leishmania colombiensis*, which is found in Colombia, Panama, and Venezuela and has been seen in sporadic human cases.

The species of subgenus *Leishmania* (Saf Janova, 1982) includes the following: *Leishmania amazonensis*, which is responsible for the anergic diffuse cutaneous form and the cutaneous forms with disseminated lesions; *Leishmania chagasi*, which causes visceral American leishmaniasis and has a wide distribution in Latin America, extending from Mexico to Argentina; *L. mexicana*, which is observed in Mexico, Colombia, the Caribbean Sea region, and Ecuador and produces muco-cutaneous leishmaniasis (“Espundia”) and the classic cutaneous form known as chiclero’s ulcer (gum tree harvester’s ulcer); *Leishmania pifanoi*, which is the causal agent of muco-cutaneous leishmaniasis in Venezuela; *Leishmania venezuelensis*, which is observed in the Venezuelan Andes; *Leishmania donovani*, which is responsible for VL in the Old World; *L. infantum*, which causes VL and CL in infants; *L. tropica*, which is the causal agent of CL; and *Leishmania aethiopica*, which is the causal agent of CL and muco-cutaneous leishmaniasis ^32^.

The duration of the life cycle in the vector varies from 4 to 18 days, depending on the species of *Leishmania*; it can be extended at low temperatures or shortened at high temperatures ^7^.

In Mexico, some rodent species have been identified as reservoirs for the parasites. For a species to be considered a reservoir, it must fulfill two criteria: (1) it must carry enough parasites to make it an effective vector at the moment of feeding and (2) in these species the infection must be relatively non-pathogenic or asymptomatic so as not to affect the survival of the reservoir ^22^. In these mammals, the skin and the blood provide adequate environments for the parasite to reproduce.

Laboratory studies from Ethiopia suggest that an *L. donovani* parasite load of 20,000 per mL of blood in the host is required to infect the sand fly species, such as *Phlebotomus orientalis* ^30^.

Generally, more than half of reservoirs remain asymptomatic ^22, 33^. It is possible that dogs play some role in maintaining endemic peri-urban areas; in the border strip of Mexico and Belize and Guatemala, when workers’ dogs keep company with their masters in the woods, sometimes they develop ulcers that clinically resemble the lesions of leishmaniasis ^17^.

Human-to-human transmission (mediated by vectors without any other living species) has been well documented in some endemic areas; this means that a sand fly bites the human patient and then infects another human in an “anthropozoonotic” cycle ^3, 4^.

Moreover, the demonstration of parasite in the blood of domestic animals using molecular tools such as PCR has suggested that domestic animals could serve as an alternate reservoir for an infection in India. Domestic animals like pets or cattle could increase transmission pressure by virtue of being an untreated reservoir for the parasite, and their proximity to human owners may also increase the transmission ratio because of increased availability of blood meals for the sand fly as well as organic manure for breeding of larvae and resting. In mixed dwellings where cattle sheds are attached to the house, bovine origin represented 66% of the blood meals of the sand fly whereas human blood represented 19%; however, even though domestic animals are seen to be infected, there is not enough evidence of their role in anthroponotic transmission in India and the Indian subcontinent ^30^. Such a biological cycle has never been documented in the Yucatán Peninsula ^3, 4^. Factors that affect the occurrence of disease include the agent (species of *Leishmania*), the host (genetic susceptibility, degree of immunocompetence, poor nutrition, and other underlying diseases), and environment ^4^.

Hypotheses that explain these variations include (a) differences in the virulence of the parasites, (b) differences in the cutaneous permeability, (c) individual variations of genetic susceptibility of the host, and (d) variations in the attraction of the *Phlebotomus* toward different individuals ^3, 4^.

Parasite transmission from the sand fly to the host depends on how infective the host is, how infective the sand fly is with a single bite, the average rate of biting, and the number of sand flies present. The biting rate was calculated as 0.25 per day—the inverse of the feeding interval (4 days)—and the latency period in the sand fly was 5 days ^30^.

When a human enters the jungle and is bitten by the infected *Phlebotomus*, the biological cycle, which is represented by the equation “wild animal ‘reservoir’ → *Phlebotomus*-infected female → wild healthy animal”, is broken, converting it into “wild animal reservoir → *Phlebotomus*-infected female → healthy human” ^32^.

The causal agent is an obligatory unicellular protozoan of the family *Trypanosomatidae*, suborder *Trypanosomatina*, and the genus *Leishmania.* Two stages in the life cycle have been defined: a promastigote (flagellated) that measures 12 to 20 μm is found in arthropods (diptera), which represent the vector that is infected while nourishing from the blood of the vertebrate host. It remains in the gut of the diptera where, within 4 to 25 days, it shows a second stage as an amastigote (non-flagellated); this is an obligatory intracellular parasite that measures from 2.5 to 3.5 μm and is localized to the phagocytic cells of the host. The infected female *Phlebotomus* (which is hematophagous), when biting the infected animal, absorbs the amastigote forms from the blood of the reservoir. In the insect’s gut, the parasite begins a process of transformation and the amastigotes change to procyclic promastigotes and then to metacyclic promastigotes; the latter have infectious capacity, and, upon biting a healthy animal, the vector inoculates it by regurgitating such promastigotes ^1, 4, 8, 32^.

When the parasite is deposited by regurgitation in the host tegument, it is phagocytized by local macrophages and epidermal Langerhans cells without the involvement of circulating monocytic cells, so this is a local event only in the skin, constituting localized CL (LCL).

The host machinery that mediates amastigote uptake is poorly understood; however, it is known that, in the human host, amastigotes bind Fc receptors and enter macrophages primarily through immunoglobulin-mediated phagocytosis. Wetzel *et al*. demonstrated that a tyrosine kinase non-receptor known as Abl2 facilitates *L. amazonensis* amastigote uptake by macrophages ^34^. Once inside the phagolysosomes, promastigotes differentiate into amastigotes and proliferate extensively by binary fission, evading the immune response. The biological cycle described is performed in 53 to 100 days ^1, 8, 9^. Pathogen species for humans are classified according to their molecular biology, and in America the predominant complexes are *L. mexicana* and *L. braziliensis*. In the Old World, the disease is caused by *L. tropica*, *L. major*, *L. aethiopica*, *L. donovani*, and *L. infantum*. According to the type of immune response elicited, the disease can be localized; it can have a tendency to be spontaneously healing or generalized and progressive ^35^.

Different factors that determine the parasite’s virulence have been identified experimentally and may help to understand the mechanisms of evasion of the immune response by the parasite. These can be classified into three main categories: (1) invasive and evasive determinants, such as lipophosphoglycans (not found in *L. mexicana*), leishmanolysin, and proteasomes; (2) pathoantigenic determinants such as histone “chaperones” or proteasomes; and (3) protective determinants, which are still being identified. On the other hand, it has been recognized that different components in the saliva of the *Phlebotomus* determine the local reaction after the bite.

The immunological basis of these findings is not completely understood; however, it is known that an adaptive change in the immune response occurs with a shifting from Th1 to Th2 with increments in the production of interleukin-4 (IL-4) and IL-6 or rather through inhibition of tumor necrosis factor alpha (TNFα), interferon gamma (IFNγ), IL-12, and the production of nitric oxide ^36^.

The immune response can vary depending on each clinical form. In the LCL forms, humoral immunity is not triggered; in the muco-cutaneous form, specific IgG antibodies can be detected; and in the diffuse cutaneous form, high levels of IgA are occasionally detected. ****Production of IL-4 in the first few weeks after disease onset has been observed; faster resolution of the lesions is mediated by the production of IFNγ by CD8 T lymphocytes ^35^. It was also observed that the immune response of “resistance” to infections caused by *L. major* is mediated mainly by a Th1 response with the activation of macrophages, in which the production of IFNγ predominates and prevents recrudescence and the development of chronic clinical forms.

On the other hand, progression of the disease has been associated with a Th2 type response and greater production of IL-4, IL-5, transforming growth factor beta (TGFβ), and IL-10. Similar studies have found an increment in the production of IL-1α, IL-6, IL-10, TGFβ, IFNγ, and TNFα during the early lesions of LCL, but in the chronic lesions cytokines are decreased ^37– 39^. In cases of VL, abnormal patterns of resistance, which were related to class II molecules of the major histocompatibility complex, have been identified. In cases of leishmaniasis caused by *L. major*, it has been observed that natural killer cells play a role in the initiation of differentiation of T CD4 lymphocytes and in the control of the initial response ^35^. During VL, intracellular parasites are managed via the activation of Th1-associated inflammation. IL-10, and eventually programmed death 1-mediated T-cell exhaustion, diminishes pathology caused by inflammation. It is not known which cell types are responsible for initiating T cell–produced IL-10 during VL; however, the population of IgD (hi) B cells was found to grow threefold during the progression of VL ^40^.

In both forms, there is an association between CD4 ^+^ Th1 lymphocytes with resistance and Th2 with susceptibility. Nevertheless, it is clear that CD4 as well as CD8 lymphocytes are activated and required to control the disease ^35^.

Because of these observations, it can be said that the “spectral” behavior of leishmaniasis is similar to that of leprosy in such a way that LCL represents the hyperergic pole in which patients have an intense cellular response and at the other end of the spectrum is diffuse CL with a progressive course and usually a fatal outcome.

These two ends of the spectrum yield a positive and negative response, respectively, to the leishmanin skin test (intradermal Montenegro’s reaction). The muco-cutaneous form is a variant of the hyperergic spectrum always produced by *L. braziliensis* and by definition is not part of the spectrum of the cutaneous forms ^1, 4^.

## Classification and clinical picture

The clinical features vary depending on the parasite’s characteristics and on the genetic aspects of the host that determine the effectiveness of the immune response. According to the clinical manifestations, it can be divided into (1) cutaneous (localized and disseminated), (2) muco-cutaneous, and (3) visceral or kala-azar ^2^.

## Localized cutaneous leishmaniasis

Pure CL was first described in the Old World by Lewis and Cunningham in 1876. It is caused by *L. tropica* ^17^. In the Mexican Southwest and at its border with Guatemala, the causal agent is *L. mexicana* ^2^. It occurs in areas of the body exposed to insect bites; in decreasing order of frequency, the most involved regions are the ears (areas usually involved are the helix and anti-helix), nose, upper lip, cheeks, legs, hands and forearms, and ankles ^17^. It is striking that in Guatemala the most affected sites are the upper limbs (up to 43% of cases) ^3^. The incubation period is from 1 to 4 weeks ^2^ but can last for up to several years ^1^. Patients may refer to previous travel to endemic zones ^2^.

**Figure 2.**
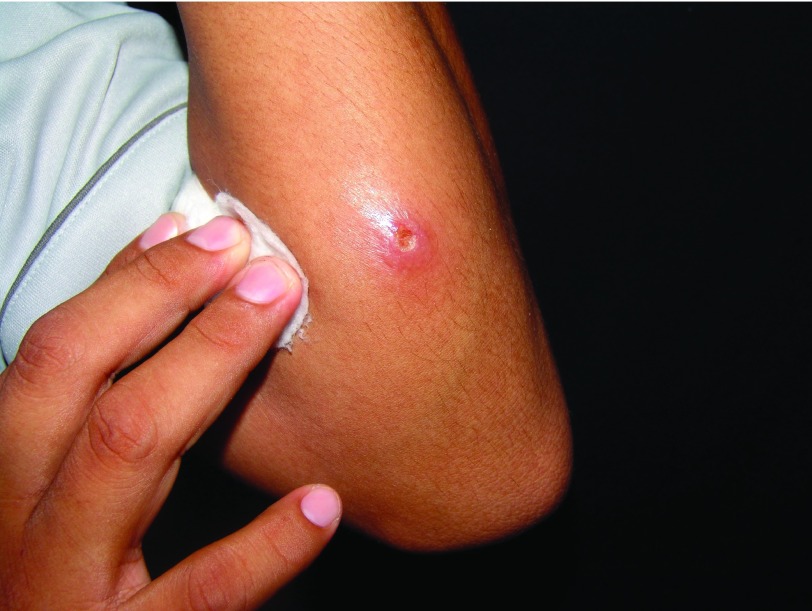
Early ulcer on the forearm with meliceric crust.

**Figure 3.**
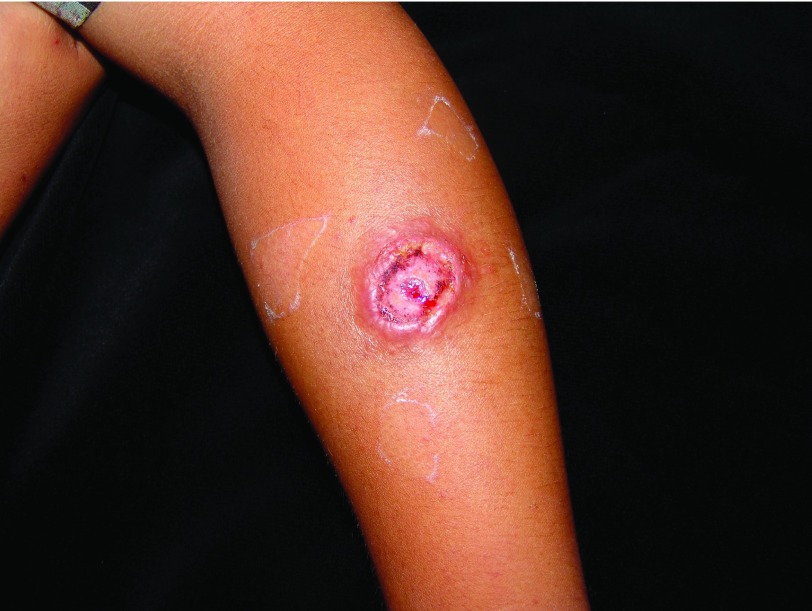
Ulcer on the upper limb with crusts and raised borders.

It is characterized by a local increase in temperature and swelling. An erythematous asymptomatic papule appears at the site of the bite, although pruritus may be present. The size ranges from 1 to 10 mm in diameter. After 2 days, it turns into a vesicle and later into a pustule, and when it breaks, either spontaneously or by trauma due to scratching, it results in a rounded ulcer with nodular or thick borders with sharp and peaked edges ( Figure 2). Such ulcers can last from 3–5 months to 15–20 years. The bottom of the ulcer shows granulation tissue that bleeds when rubbing and a pink periphery and sometimes is covered by a whitish pseudo-membrane ( Figure 3). In some cases, abundant secretion forms an adherent crust ^17^. The lesion is not painful if it is not secondarily infected. Ulcers may be solitary or multiple; autoinoculation has been observed with the infection at sites distant to the previous mosquito bite (as in the forearms) by prolonged contact with ulcerated areas ^1, 41^. The clinical picture is usually afebrile with regional adenopathy ^2, 17^.

On rare occasions, the initial lesion may not ulcerate and develop vegetating appearance ^12, 28^. LCL can heal spontaneously in 3–9 months in the case of *L. mexicana*, 2–6 months in the case of *L. major*, and 6–15 months if the agent is *L. braziliensis*, *L. tropica*, or *L. panamensis* ^4, 42, 43^.

**Figure 4.**
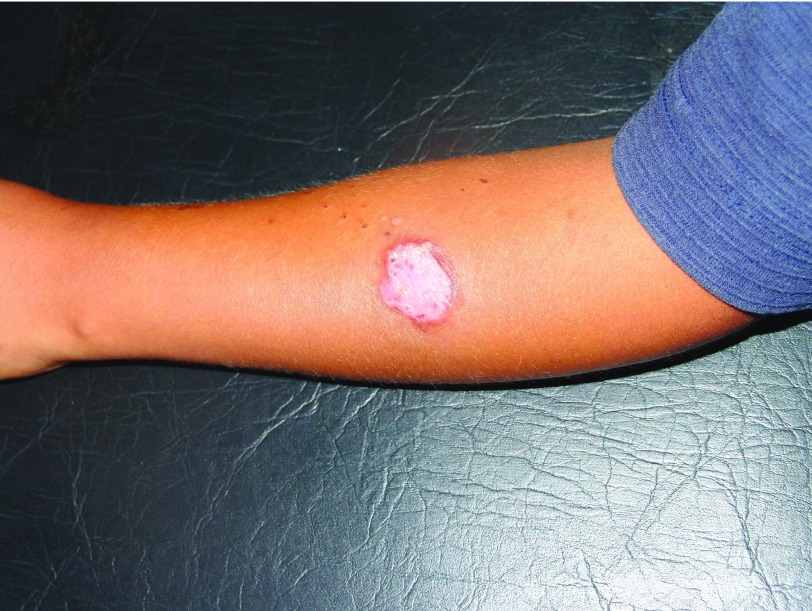
Atrophic scar on forearm.

**Figure 5.**
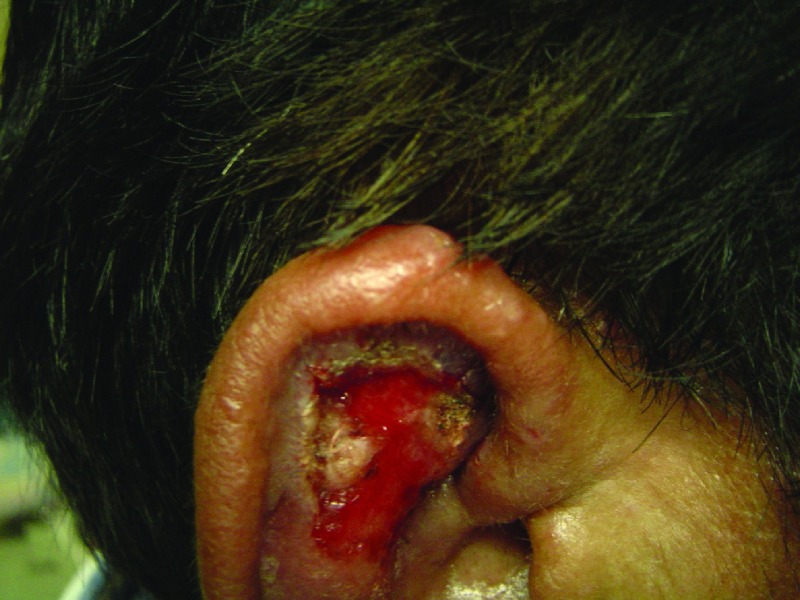
Atrophic stage of chiclero’s ulcer with deforming scarring of the ear.

**Figure 6.**
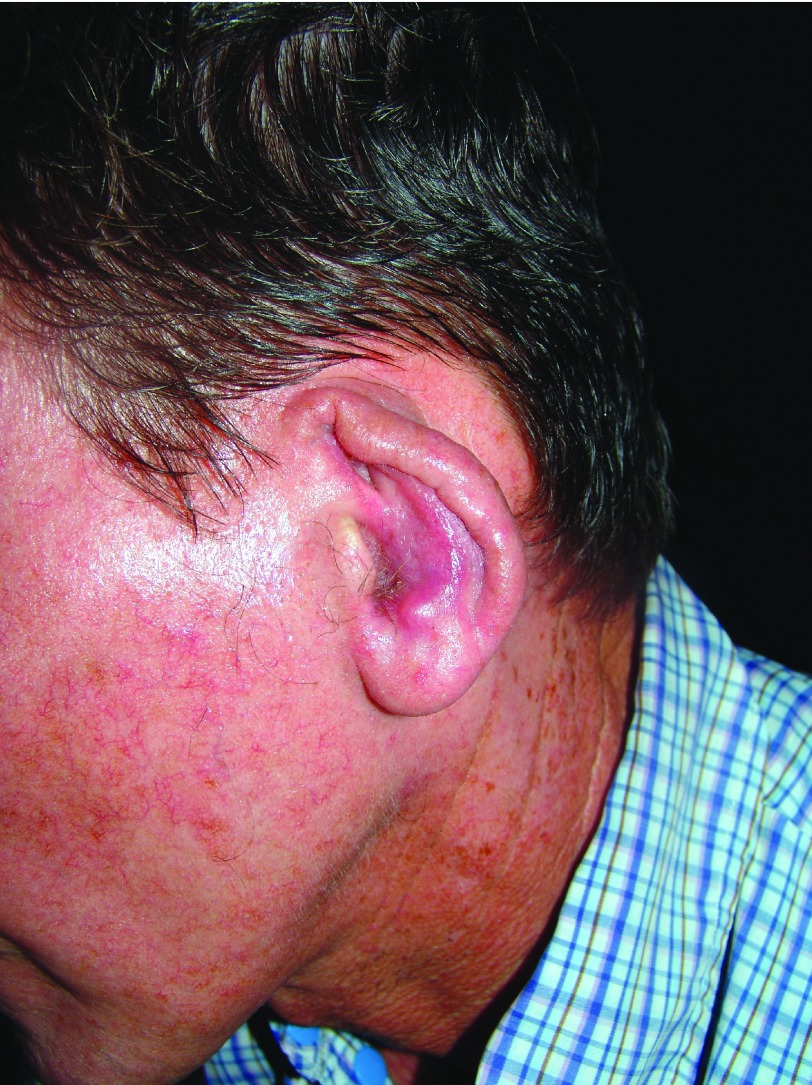
Chiclero’s ulcer with bleeding bed and periphery covered by fibrin.

Spontaneous healing in up to 4 years, in which healing progresses from the periphery to the center of the lesion, has been described ^17^. Spontaneous healing leaves a depressed plaque with uneven pigmentation and telangiectasia, or retractile scars with hypopigmented center and hyperpigmented periphery, as well as local deformity due to large tissue destruction ( Figure 4). When the ear is affected (chiclero’s or gum tree harvester’s ulcer), it results in mutilations in the form of notches, or clefts ^1, 17, 41^ ( Figure 5 and Figure 6).

Around 33% of patients can relapse, or the scarring lesion may reopen, presenting with features similar to those of the original episode; recurring manifestations can be mild or more severe than those observed at the initial episode ^1, 17^, called *Leishmania recidivans* ^4^. There is an abortive form in which the lesion is a “regressive” papule ^44^.

## Diffuse cutaneous leishmaniasis

**Figure 7.**
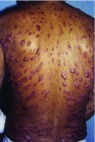
Diffuse cutaneous leishmaniasis (anergic clinical form).

This form is characterized by anergy (that is, lack of cellular immune response to parasite antigens). This allows dissemination through tissue, lymph, and blood pathways, developing lesions in most of the skin, except in the scalp, and sometimes with involvement of mucous membranes. It is observed in Central America, Amazonian Brazil, Venezuela, Ethiopia, and Kenya; is caused by the *L. mexicana* complex ( *L. amazonensis*, *L. braziliensis* and *L. pifanoi*); and predominates in exposed areas (for example, the ears (pinna), cheeks, and extremities). It usually starts with hard erythematous nodules and reddish-brown infiltrative smooth or verrucous plaques. The latter may or may not ulcerate and are present first on the face and afterwards progressively affect the extremities, buttocks, and mucous membranes and in some cases can involve the entire skin surface ( Figure 7). Lymphedema, lymphadenopathy, poor general condition, and fever may be observed. When the mucosae such as the oropharynx and nasopharynx are involved, painful nodules may lead to airway obstruction. This clinical form is very difficult to treat, there is no spontaneous resolution, and a long evolution of up to 20 years has been observed ^1, 4, 9^.

## Muco-cutaneous leishmaniasis, cutaneous-mucosal, American cutaneous, or “Espundia”

**Figure 8.**
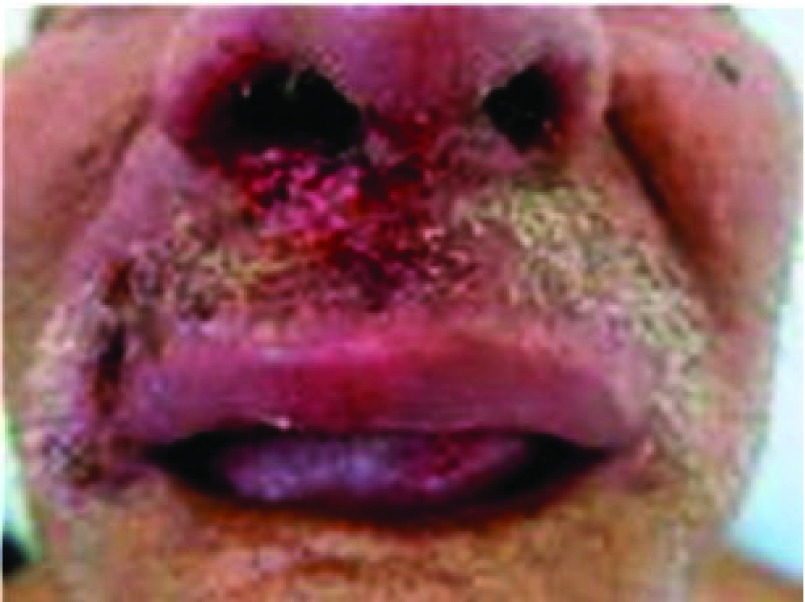
Muco-cutaneous leishmaniasis (“Espundia”).

In South America and in endemic areas, it has been noted that in 1 to 10% of patients the LCL form evolves into muco-cutaneous leishmaniasis after 5 years of having healed. Cases from Bolivia, Brazil, and Peru represent 90% of this variant. The causal species of this clinical form belong to the complex *L. braziliensis*, which includes *L. braziliensis*, *L. guyanensis*, and *L. panamensis*. It causes invasion and destruction of the nasopharyngeal mucosa ^45^ ( Figure 8). Invasion occurs slowly, sometimes causing no initial disturbance, thus allowing the mucosal injury to go unnoticed; on some occasions, it causes only mild local pruritus and swelling ^4, 9, 17, 42, 46^. In Panama, the “Bejuco’s ulcer” affecting young individuals is caused by *L. panamensis*.

Usually, lesions start in the nasal mucosa and spread to the oral and pharynx mucosa, the larynx, and the skin of the nose and lips ^1^. Lesions of the oral mucosa usually produce symptoms that range from simple discomfort and mild pain or odynophagia to cachexia in extreme cases; the latter is observed only in cases in which the lesion involves the totality of the pharynx, larynx (with hoarseness), and esophagus (with dysphagia). Early in the disease, there is infiltration of the mucosa with superficial ulcerations; later on, when the ulcers are well developed, their borders have a necrotic appearance and are torn and detached. The uvula, pillars of the palate roof, and tonsils can be destroyed. Owing to superimposed infections, the regional lymph nodes can become infarcted and become painful. When the infection is in the nasal cavity, atrophy of the nasal turbinates and destruction of the cartilaginous septum with foul smell can occur and in extreme cases can cause death ^4, 17, 46^.

## Visceral leishmaniasis, or kala-azar (black fever)

The febrile infectious illness known as kala-azar (black fever) spreads over a large part of south and east Asia (mainly in India and China), a large part of Africa, the Mediterranean (affecting children and adults), and South America (where children are affected). It is caused by *L. donovani* (India and Eastern Africa), *L. infantum* (Mediterranean area), and *L. chagasi*, *L. amazonensis*, and *L. tropica* in South America ^47, 48^. The incubation period is from 3 to 8 months. The at-risk population includes preschool children and immunocompromised and undernourished individuals.

Recently, this type of leishmaniasis has been seen with increasing frequency in patients who have AIDS or who are intravenous drug users or both, suggesting a potential transmission mechanism through contaminated syringes ^1^. Lesions in the reticuloendothelial system may follow a subclinical course (the minority of cases), or patients may be oligosymptomatic or frankly symptomatic. It is manifested by lymphadenopathy, hepatomegaly, splenomegaly, pallor, anemia, leukopenia, thrombocytopenia, fever, night sweats, weakness, anorexia, asthenia, cutaneous pigmentation, and weight loss, which can progress rapidly in weeks or months. In addition, affected children present characteristic chronic diarrhea and growth retardation.

Typical laboratory abnormalities include pancytopenia and hypergammaglobulinemia. If the disease progresses in the absence of treatment, cachexia and multisystem failure, hemorrhage due to thrombocytopenia, and superimposed infections can cause death.

A decrease in the number or function of CD4 cells may cause recurrence, as in the case of patients receiving corticosteroids or chemotherapy, in transplant recipients, or in association with infection with HIV; in this scenario, a new therapeutic regimen may be required ^49– 51^.

## Leishmaniasis and HIV

Initial data in this regard came from Southern Europe from intravenous drug abusers who share syringes, leading to a decrease in the number of T CD4 lymphocytes; in 90% of these patients, the infection represents reactivation of a previously acquired subclinical or latent infection. This immunodeficiency, in turn, leads to a lower response to treatment and frequent recurrence. No guidelines have been established to discontinue anti-parasite medications after the T CD4 cell counts increase post-treatment with the highly effective anti-retroviral treatment ^49, 52^. Similar to HIV-infected patients, pregnant women can be considered a particular population with a condition predisposing to *Leishmania* reactivation and to changes in immune response owing to a switch toward a Th2 response that has been reported during pregnancy ^53^.

## Diagnosis

Diagnosis is based on the clinical and congruent epidemiological context ^17^. Laboratory confirmation and identification of the species of *Leishmania* are important ^2^. The protozoan is found in the scraping of cutaneous or mucosal ulcerations (especially scraping the borders) as well as in non-ulcerated lesions ^17^. Biopsy is another diagnostic tool; it should be obtained from the active border of the lesion. A smear may reveal the parasites in free form or inside macrophages or less frequently in polymorphonuclear leukocytes, ranging in number from 2 to 20 in a single cell. The parasite shape is oval or piriform (with an oval or rounded nucleus) and ranges in size from 2 to 5 μm long and 1 to 2 μm wide. Exceptionally flagellated forms have been observed. Inoculation is also used for the culture ^1, 17, 41^.

## Histopathological data

In cases of LCL, sections stained with hematoxylin and eosin show epidermal atrophy or hyperplasia with an inflammatory infiltrate consisting of macrophages, lymphocytes, and plasma cells with focal necrotic areas. In the early stages of the infection, parasites can be recognized within cytoplasmic vacuoles in histiocytes (Leishman bodies) ^2^, and in late stages, infected macrophages are less numerous, with few amastigotes predominating, and with some lympho-histiocytic infiltrates confirming a tuberculoid granuloma. The granuloma evolves in three phases: (1) development of a mononuclear phagocytic infiltrate, (2) maturation and cellular aggregation into a disorganized granuloma, and (3) maturation of these cells into an epithelioid granuloma ^54^. Patients with diffuse CL have lesions with highly parasitized macrophages and few dermal lymphocytes ^1, 2^.

Based on the Magalhaes histopathological classification, the findings are categorized into five groups: type I, which exhibits an exudative cellular reaction due to lymphocyte, histiocyte, and plasma cell infiltration without granuloma; type II, which exhibits exudative necrotic reaction characterized by cellular infiltration and necrosis without granulomas; type III, which has disorganized granulomatous necrotic and exudative granuloma corresponding to the pattern of a chronic and necrotic inflammatory reaction; type IV, which shows an exudative granulomatous reaction without necrosis and with disorganized granulomas; and type V, which exhibits an exudative tuberculoid reaction with typical organized tuberculoid granuloma. Of these types, the most frequently encountered and documented in patients with chiclero’s ulcer has been type IV ^54^. When special stains such as Wright-Giemsa are used, the protozoan cytoplasm stains blue and the nucleus stains red ^17^.

## Cultures

The culture is performed through the inoculation of the triturated tissue in special media. *Leishmania* is cultured in Novy–McNeal–Nicolle medium (currently called “N-N-N medium”) or in a variant of the Evans biphasic medium. In the first culture, forms devoid of flagella are almost always obtained, but from the first re-seeding onwards flagellated forms appear which reach up to 50 μm long ^17^. Both techniques (microscopic exam and culture) have a sensitivity of 85% ^49, 55^.

## Laboratory data

The Montenegro skin test is sensitive and specific. It is positive for the localized forms and negative in the anergic forms; a positive test supports the diagnosis (especially when the patient does not live in endemic areas), but a negative test does not exclude it ^1^. This allergic index is useful to determine whether previous contact with the parasite has occurred, even in the absence of lesions ^19^. A positive skin test is when the reaction is greater than 5 mm after 72 hours ^18^. However, intradermal injection of *Leishmania* antigen is not approved by the US Food and Drug Administration ^56^.

The parasites are observed in the smear or in the imprint stained with Giemsa or Wright; this is the most common and useful diagnostic method ^4, 18^. In the case of the visceral forms, the diagnosis is performed by observing protozoans through the microscope in a tissue sample obtained by aspiration from the spleen, bone marrow, or lymph node with a sensitivity of 95%, 55–97%, or 60%, respectively.

Determination of serum anti- *Leishmania* IgG and the determination of anti-K39 antibodies are also used, with a sensitivity of 90 to 100% and variable specificity according to geographic area. Detection of *Leishmania* antigens in urine is a new diagnostic approach ^49, 57^.

The complement fixation test is used to detect antibodies; titers equal to or above 1:8 are consistent with infection, as are titers equal to or above 1:16 in immunofluorescence tests. However, lower titers do not exclude the infection ^9^. In addition, antibodies can be detected by direct agglutination, direct immunofluorescence, and enzyme-linked immunosorbent assay ^1, 2^; however, these serological tests are infrequently recommended in CL because of their scarce availability ^18^.

PCR has a reported specificity in LCL of 100% with an improved sensitivity of 20 to 30% when compared with conventional parasitology diagnosis; it can also be used in cases of mucosal leishmaniasis. Unfortunately, this method is available only in specialized laboratories or in travelers’ clinics ^4^.

Recently, in a study in Jordan conducted by Hijjawi *et al*. using ITS1-PCR applied to 41 skin scraps on glass slides, 30 were successfully identified ^58^. Furthermore, in 28 samples, PCR-RFLP analysis allowed species identification corresponding to *L. major* and two more corresponding to *L. tropica*. However, it is still not available in medical laboratories in Jordan. For the most part, molecular amplification of the hsp70 gene fragment (PCR-hsp70) followed by RFLP analysis is a valid tool for the identification of *Leishmania* species isolated from clinical samples of patients, as was demonstrated in Colombia by Montalvo *et al*., and could be applicable to epidemiological studies ^59^.

## Prophylaxis

The eradication of the vector through insecticides, elimination of stagnant water, use of insect repellents, and prophylaxis can be achieved through the use of thick clothes with long sleeves that can be impregnated with insecticides ^4^ and long pants and by avoiding night walks in jungle areas ^17^. The WHO is planning a vaccine that would protect against all types of leishmaniasis ^44^.

## Treatment

The only effective treatment with satisfactory clinical and microbiological results for all clinical forms of leishmaniasis is achieved with current intravenous pentavalent antimonials (Sb5+) in the form of sodium stibogluconate (SSG; Pentostam, UK) or meglumine antimoniate (Glucantime, France), except in the state of Bihar in India ^49^. In Bihar, parasite resistance to antimonials caused a dramatic rise in treatment failure of up to 65% between 1980 and 1997; in addition, there is the potential for resistance to miltefosine and liposomal amphotericin B to develop ^30^.

It has been generally accepted that American CL, especially that caused by *L. braziliensis*, should be treated with a dose of 20 mg of Sb5+/kg per day for 20 days, especially to prevent disfiguring sequelae in mucosal lesions ^17, 60, 61^.

Trivalent antimonials are prescribed parenterally (repodral and anthiomaline) in a dose of 2–3 mL (0.02 to 0.03 g) on alternate days for 12 to 20 days, pentavalents such as meglumine (glucantime) in a dose of 10–60 mg/kg for 12 days to 3 weeks (or until clinical and parasitological evidence of cure), and SSG (Pentostam) in a dose of 20 mg/kg per day for 30 days, not necessarily consecutive; this periodicity can be determined by the onset of side effects. The drug should be diluted in 200 mL of 5% dextrose and delivered in 1 hour ^32^. Intravenous administration is used only in hospitalized patients, as intramuscular injection is very painful, but it is the only feasible alternative for treatment on a large scale.

Intralesional treatment has been documented recently as the first therapeutic line in LCL caused by *L. major*, *L. tropica*, and *L. panamensis*. It causes damage to the cellular membranes and fragmentation of microtubules, preventing division of the organism. On the other hand, previous experimental studies have shown that the combination with lidocaine, besides decreasing local discomfort, favors the fragmentation and loss of morphologic definition of the plasma membrane (due to its amphophylic properties) and other organelles in the protozoa of *L. braziliensis* ^4, 62^.

The most frequently described adverse effects of antimonials are local irritation, anorexia, nausea, vomiting, myalgia, arthralgia, increases in hepatic enzymes, urea, and creatinine, and electrocardiographic alterations, such as inversion of the T wave, prolongation of Q-T segment, depression of the S-T segment, and sinus bradycardia. In case these effects occur, it will be necessary to stop the drug until normalization of the clinical and laboratory abnormalities, re-introducing the drug on alternate days ^2, 32^.

In the diffuse form, pentamidine is useful and is the second choice. Each flask has 300 mg of the drug, a dose that should be diluted in 5 mL of distilled water to be applied intramuscularly in the gluteus. The dose is 4 mg/kg with a maximum dose of 240 mg per day on alternate days. The total dose is dependent on the clinical response and the adverse effects, including nephrotoxicity, hepatotoxicity, hypertension, hypoglycemia, hyperglycemia (rarely), electrocardiographic alterations, gluteal abscess, central facial paresthesia, cephalea, epigastralgia, and vertigo.

For treatment of the muco-cutaneous forms, the duration of a parenteral therapeutic regimen with antimonials is 28 days, achieving a cure rate of about 75% in patients with mild or moderate forms; it is lower in patients with more severe clinical forms, in whom amphotericin B is used as a rescue treatment, and in patients with anergic forms ^49, 63^.

Treatment failure for VL with pentavalent antimonial (SSG) has been reported in recent years in Nepal; as a result, liposomal amphotericin B is currently recommended by the National Program of Nepal for kala-azar treatment ^15^.

The drug also results in a good response in immunocompromised patients, but the percentage of recurrences in these patients is high. Amphotericin B is an extremely effective but toxic alternative; it is effective even in forms resistant to antimonials. The dose is l mg/kg per day with a maximum of 50 mg per dose. It is prepared by diluting it in 500 mL of 5% dextrose and delivered on alternate days up to a total dose of 1 to 1.5 g, while liposomal amphotericin is used at 2 to 3 mg/kg as a total dose over the course of 20 days.

Clinical improvement is observed from 7 to 10 days after therapy is initiated; after 2 weeks of treatment, the clinical cure is almost complete with cessation of fever, decrease in size of the spleen, and absence of amastigotes in the aspirate.

An alternative is the transfer factor and more recently paromomycin, which has finished phase III experimental studies in India and Africa and has been approved in some cases ^49, 64^. In some cases of CL, diaminodiphenyl sulfone (Dapsone ^®^) has been useful at a dose of 3 mg/kg per day for 3 weeks ^1, 2, 32^.

In cases caused by *L. mexicana*, there is a good response to ketoconazole 200 to 400 mg per day for 4 to 6 weeks with or without cryotherapy ^2, 65^ or itraconazole 200 to 400 mg per day for 1 to 2 months ^32^. Fluconazole has not been demonstrated to be effective as a therapeutic option ^66^.

Other alternatives include rifampicin 600 to 1,200 mg per day for more than 2 months, either alone or with isoniazid, IFNγ at a dose of 50 to 100 μm/m ^2^ per day (subcutaneously for 10 to 15 days, although it is not curative if used alone), recombinant human granulocyte-macrophage colony-stimulating factor (rHGM-CSF) at subcutaneous doses of 5 μg/kg per day for 10 days or intralesional 200 μg per dose, metronidazole 250 mg three times a day in cycles of 10 to 15 days, trimethoprim-sulfamethoxazole 160/800 mg twice a day for 4 weeks, azithromycin at a dose of 500 mg per day for 3, 5, and 10 days or 1 g per day for 2 days, and pentoxifylline in doses of 1,200 mg per day for 30 days ^32^.

Clinical studies with paromomycin and miltefosine have shown them to be useful; currently, the combination of thermotherapy and miltefosine has been considered as an effective alternative in the treatment of CL ^4, 67, 68^.

A randomized phase II–III clinical trial conducted in Brazil with 90 patients with confirmed CL due to *L. guyanensis* to compare miltefosine versus parenteral antimonials revealed cure rates of 71.4% and 53.6%, respectively, with no statistically significant differences between age groups within the same treatment arms ^69^. However, in a phase II open-label, non-comparative randomized trial conducted in Sudan and Kenya to evaluate the efficacy and safety of three treatment regimens for VL caused by *L. donovani* based on the combination of liposomal amphotericin, SSG, and miltefosine divided into three arms of 50 patients each, the results demonstrated cure ratios of 87% for liposomal amphotericin plus SSG, 77% for liposomal amphotericin plus miltefosine, and 72% for miltefosine alone, and efficacy was lower in younger patients; therefore, none of these regimes achieves cure ratios of 90%, so they should not be used as monotherapy ^70^.

Locally, one can use antiseptics such as 15% paromomycin sulfate and 12% methylbenzethonium ointment twice daily for 10 days to 3 weeks. Another alternative in the local treatment of LCL is the injection of intralesional amphotericin, which has demonstrated its effectiveness using 2.5 mg/mL weekly without recurrences after a 6-month period of follow-up in a study performed by Mushtaq *et al*. in India ^71^. Thermotherapy, cryosurgery, curettage, and laser and radiotherapy have been tried with relative efficacy ^1, 4, 72, 73^. Sometimes, in spite of successful treatment, recurrent lesions have been reported ^74^. Immunotherapy that combines topical imiquimod with systemic antimonials and vaccines against the sand fly will be fundamental for the future treatment and prevention of leishmaniasis ^75^.
